# Biological Activity of *Humulus lupulus* (L.) Essential Oil and Its Main Components against *Sitophilus granarius* (L.)

**DOI:** 10.3390/biom10081108

**Published:** 2020-07-25

**Authors:** Gianluca Paventi, Laura de Acutis, Antonio De Cristofaro, Marco Pistillo, Giacinto S. Germinara, Giuseppe Rotundo

**Affiliations:** 1 Department of Medicine and Health Sciences “V. Tiberio”, University of Molise, via de Sanctis, 86100 Campobasso, Italy; 2 Department of Agricultural, Environmental and Food Sciences, University of Molise, via de Sanctis, 86100 Campobasso, Italy; laura_de_acutis@yahoo.it (L.d.A.); decrist@unimol.it (A.D.C.); 3 Department of the Sciences of Agriculture, Food and Environment, University of Foggia, Via Napoli 25, 71122 Foggia, Italy; marco.pistillo@unifg.it

**Keywords:** botanical insecticides, hop terpenes, pest control, acetylcholinesterase, electroantennography

## Abstract

Besides its use in the brewing industry, hop cones appear as a powerful source of biologically active compounds, already checked for their putative anticancer, antimicrobial, and other bioactivities. Conversely, hop use in pest control remains to date under-investigated. Therefore, the biological activity of hop essential oil (EO) and its main constituents was investigated here against *Sitophilus granarius*. Adult contact toxicity was found 24 h after treatment with hop EO (LD_50_/LD_90_ 13.30/40.23 µg/adult), and its three most abundant components, α-humulene, β-myrcene, and β-caryophyllene (LD_50_/LD_90_ 41.87/73.51, 75.91/126.05, and 138.51/241.27 µg/adult, respectively); negligible variations at 48 h, except for α-humulene (LD_50_/LD_90_ 26.83/49.49 µg/adult), were found. The fumigant toxicity of the EO and terpenes was also checked: in the absence of wheat grains, β-myrcene showed the highest inhalation toxicity (LC_50_/LC_90_ 72.78/116.92 mg/L air), whereas α-humulene, β-caryophyllene, and the EO induced similar values (LC_50_/LC_90_ about 130/200 mg/L air); with the exception for EO, the wheat presence increased (30–50%) LC_50_/LC_90_ values. Moreover, EO and terpenes were perceived by insect antennae and elicited repellent activity. Only β-caryophyllene showed an anticholinesterase effect, this suggesting that different mechanisms of action should be responsible for hop EO toxicity. Therefore, hop EO appears suitable for developing control means against this pest.

## 1. Introduction

Hop, *Humulus lupulus* (L.), is a flowering and perennial plant belonging to *Cannabacea* family, whose female flowers, clustered in inflorescences, are used in the brewing process of beer. However, the aerial part of this plant produces several compounds as bitter resins, essential oil (EO), tannins, and terpenes, proved to have biological activities. For instance, xanthohumol was reported to exert anticancer [1,2], antioxidant [3], and antimicrobial [4] effects; hop iso-α-acids positively affect glucose metabolism, diet-induced obesity, and its relative cognitive decline in rodents [5,6,7,8]; in addition, the main components of the EO, such as α-humulene, α-myrcene, and β-caryophyllene, showed activities against different strains of Gram-positive and Gram-negative bacteria [9]. Moreover, besides medical purposes (see for other refs [10,11]), the interest in this plant is increasing in the light of its possible use also in pest control. Recent papers, in fact, highlighted activities of hop against detrimental fungi [12], mites [13], and insects [14,15]. This aspect appears extremely urgent since, as known, the traditional control of stored-product insect pests has relied primarily on synthetic insecticides like organochlorines, organophosphates, methyl bromide, and phosphine [16]. However, the indiscriminate use of these chemicals has raised long-term human health and environmental concerns, mainly due to their slow degradation in the environment and toxic residues in the products, as well as the development of resistance to pesticides in pest populations [17]. In particular, several studies indicate an increase in resistance of some stored-product insect pests to conventional synthetic pesticides [18] (for other references see Reference [19]). Moreover, the preimaginal stages of some pests, such as *Sitophilus granarius* (L.) (Coleoptera, Curculionidae), develop within grains, limiting the efficacy of these pesticides and leading to a more intense application [20]. For these reasons, the development of alternative strategies to synthetic chemicals is very important in pest control, especially in this latter case. In this view, aromatic plants appear to be a remarkable source of biologically active compounds, among which some are already used as natural insecticides [21,22].

Thus, in the present study, the attention is focused on the wild hop of the southern Italian Apennines by investigating the contact and inhalation toxicities and the repellent effects of hop EO and its main components against the granary weevil, *S. granarius*. Moreover, the electrophysiological and the anticholinesterase activities of the EO and individual compounds were also determined to contribute to the characterization of their bioactivity towards granary weevil adults.

## 2. Materials and Methods

### 2.1. Plant Material and Pure Compounds

Aerial parts of hops were collected during the flowering stage in Bojano (Molise region, Italy) at 482 m a.s.l. The area (N 41°47’840” E 14°49’428”) has an average annual rainfall of 700 mm, and the mean annual temperature of 14–15 °C. The soil where hop plants were harvested has a neutral reaction (pH 7.25), a sandy texture (fine sand 54%, coarse sand 23%), a low organic carbon content (10.7 g/Kg), and a low C/N ratio (5.9). It is also a strongly calcareous soil (CaCO_3_ 37.26%), with a very low content of available phosphorus (P_2_O_5_ 5.14 mg/kg) for plants. A voucher specimen was deposited in the herbarium at the Department of Agricultural, Environmental, and Food Sciences of the University of Molise. β-Myrcene, α-humulene, and β-caryophyllene (purity grade ≥90%, 96%, and 98%, respectively) were purchased from Sigma-Aldrich (Milan, Italy).

### 2.2. Insect Rearing

*S. granarius* were reared on wheat grains for several generations in glass cylindrical containers (Ø 15 × 15 cm) closed by a metallic net (1 mm) and maintained in the dark in a climatic chamber set at 25 ± 2 °C and 60 ± 5% r.h. Adults beetles, 2–4 weeks old, were used for the experiments.

### 2.3. Extraction of EO

Hop female flowers were oven-dried (36 ± 2 °C for 72 h), ground, and aliquots of powder (50 g) were used to obtain essential oil (EO) by hydro-distillation in a Clevenger-type apparatus for 3 h according to the method recommended in the current European Pharmacopoeia (2010). The oil was dried over anhydrous sodium sulphate and stored at 4 °C in the dark until analyzed and tested. The yield obtained was 0.82 ± 0.13% (mean ± SE, *n* = 3) and EO density was 0.875 g/mL.

### 2.4. Gas Chromatography-Mass Spectrometry (GC-MS)

The EO was diluted 1:100 with dichloromethane-hexane (2:3) and a 1 μL sample was injected in the gas chromatographic system. Three samples were analysed. A 7890B series gas chromatograph (Agilent Technologies) with an Agilent 5977 mass selective detector (MSD) and equipped with a HP-5MS capillary column (30 m × 0.25 mm i.d., 0.5 μm film thickness, Agilent Technologies, Santa Clara, CA, USA) was used. The carrier gas was helium at a flow rate of 1.25 mL/min.

The injection was made in the splitless mode, and the injector temperature was 250 °C. The column oven temperature was initially held at 60 °C, then it was programmed to 220 °C at 3.0 °C/min and to 250 °C at 15.0 °C, with a final holding time of 20 min. Spectra were recorded in the electron impact mode (ionization energy, 70 eV) in the range of 30–550 amu at 2.9 scans/sec. A solvent delay time of 5 min was used to avoid overloading the mass spectrometer with the solvent. The identification of volatile compounds was achieved by comparing mass spectra with those of the data system library (NIST08, *p* > 90%) and wherever possible by comparing retention times and mass spectra with those of commercially available standards. Moreover, a mixture of a continuous series of straight-chain hydrocarbons, C_8_-C_20_ (Alkane Standard Solution C_8_-C_20_, Sigma Aldrich, Milan, Italy) was injected into an HP-5MS column under the same conditions previously described for the oil samples to obtain the linear retention indices (RIs). The RIs were calculated according to the RI Van den Dool and Kratz equation [23]. Component relative percentages were calculated based on GC peak areas.

### 2.5. Contact Toxicity

The contact toxicity of hop EO and individual compounds to granary weevil adults was determined using topical application, as reported in previous studies [24,25]. All compounds and EO were dissolved in *n*-hexane, and for each sample two-fold serial dilutions were prepared. For each dilution, an aliquot (0.5 µL) was applied on the pronotum of *S. granarius* adults in thanatosis through a Hamilton’s syringe (700 series, Microliter^TM^ Hamilton Company, Reno, NV, USA). Each dilution was applied on 5 unsexed adults (mean body weight 1.983 ± 0.018 mg) of *S. granarius* and an equal number of individuals was treated with *n*-hexane as a control. Insects were confined to each Petri dish within metal rings (Ø 4.0 × 2.5 cm), covered with a metallic net (mesh 1 mm) to prevent insects escape, with 5 wheat kernels and maintained under controlled condition (26 ± 2 °C and 60 ± 5% r.h.) in the dark. Three replicates of each dose and control were set up. The mortality of adults was observed after 24 and 48 h. The percentages of mortalities were transformed to arcsine square-root values to meet assumptions of normality and submitted to analysis of variance (ANOVA). Treatment means were compared and separated by Tukey’s HSD test. Statistical analyses were performed with SPSS (Statistical Package for the Social Sciences) v.23 for Windows (SPSS Inc., Chicago, IL, USA).

The Lethal Dose 50 (LD_50_) and 90 (LD_90_) values, the confidence upper and lower limits, regression equations, and chi-square (χ^2^) values were calculated using probit analysis [26].

### 2.6. Fumigant Toxicity

The fumigant toxicity of hop EO, β-myrcene, α-humulene, and β-caryophyllene to granary weevil adults in the presence and absence of wheat grains was assessed using the method described in previous studies [25,27,28]. A glass container (600 mL) was used as a fumigation chamber. A filter paper (Whatman No.1) disc (Ø 2.0 cm) was suspended in the centre of the chamber by an iron wire attached to the under-surface of its aluminium screw cap. Ten adult insects were placed in the chamber, the paper disc treated with an appropriate volume of the test substances, and the glass container tightly closed. In tests with wheat grains, intact kernels (200 g) were placed on the base of the fumigation chamber together with the insects. An untreated paper disc was used as a control. Three replicates of each dose and the control were set up. Bioassays were carried out in the dark at 26 ± 2 °C and 60 ± 5% r.h. for 24 h. Dead insects were counted after exposure to fresh air in Petri dishes after 24 h. For each set of experiments, treatment means were submitted to ANOVA and separated using Tukey’s HSD test. The LC_50_ and LC_90_ values, expressed as mg/L air, the confidence limit of upper and lower confidence levels, regression equations, and χ^2^ values were calculated using probit analysis [26].

### 2.7. Two-Choice Behavioural Bioassays

The repellent activity of hop EO, β-myrcene, α-humulene, and β-caryophyllene solutions to granary weevil adults and their ability to disrupt insect orientation to odours of wheat grains were evaluated in a two-choice pit-fall bioassay similar to that described in a previous study [29]. The test arena was a steel container (Ø 32 cm × 7 cm height) with two diametrically opposed holes (Ø 3 cm) located 3 cm from the side wall. A filter paper disc (Ø 0.7 cm) was suspended at the centre of each hole by a cotton wire taped to the lower surface of the arena. Glass flasks (500 mL), assigned to collect the responding insects, were positioned under each hole. The inside necks of the collection flasks were coated with mineral oil to prevent insects from returning to the arena. Thirty unsexed insects, left for at least 4 h without food, were placed under an inverted Petri dish (Ø 3 cm × 1.2 cm high) at the centre of the arena and allowed 30 min to acclimate prior to release. During the assay, the arena was covered with a steel lid to prevent insects from escaping. In each experiment, insects were given a choice between the odours emitted by wheat grains (200 g; 14.5% moisture content) left in a collection flask alone or plus a set dose (10 µL of acetone solution) of EO or individual compounds, adsorbed onto the overlying filter paper disc, and acetone (10 µL) adsorbed onto the opposed paper disc as control. In each set of experiments, five doses (0.54, 1.09, 2.19, 4.38, 8.75 mg) were assessed for hop EO, and four doses for individual compounds: β-myrcene (0.53, 1.07, 2.14, 4.28 mg), α-humulene (0.55, 1.11, 2.22, 4.44 mg), and β-caryophyllene (0.56, 1.13, 2.26, 4.51 mg). Tests lasted 3 h and were carried out in the dark at 26 ± 2 °C and 60 ± 5% R.H. Each bioassay was replicated four times and insects were only used once. In each experiment, a response index (RI) was calculated by using RI = [(T − C)/Tot] × 100, where T is the number responding to the treatment, C is the number responding to the control, and Tot is the total number of insects released [30]. For each test stimulus, the significance of the mean RIs was evaluated by comparing the mean number of insects in the treatment and control using Student’s *t*-test for paired comparisons. The mean numbers of insects found in the treatment and in the control and the mean RIs at different doses of EO and individual compounds alone and in the presence of wheat grain odours were subjected to ANOVA and ranked according to Duncan’s MRT test.

### 2.8. Electroantennography (EAG)

To ascertain the olfactory sensitivity of male and female granary weevil antennae to increasing concentrations of hop EO and its three main components, EAG assays were performed using the technique described in previous studies [27,31]. The head of the insect was excised from the prothorax and mounted between two properly pulled (PC-10 puller, Narishige, Tokyo, Japan) glass capillary electrodes (Microglass, Naples, Italy) filled with Kaissling saline [32]. The recording electrode (diameter ~ 100 μm) was put in contact with the dorsal surface of the terminal antennal segment while the neutral electrode was inserted into the base of the head. AgCl-coated silver wires were used to maintain the electrical continuity between the antennal preparation and an AC/DC UN-6 amplifier in DC mode connected to a PC equipped with the EAG 2.0 program (Syntech Laboratories, Hilversum, The Netherlands).

Hop EO, β-myrcene, α-humulene, and β-caryophyllene solutions (50, 100, 200 µg/µL) in hexane (Sigma Aldrich, Milan, Italy) were prepared. The test stimulus (10 µL of an EO or compound solution) was adsorbed onto a filter paper (Whatman No. 1) strip (2 cm^2^) inserted in a Pasteur pipette (15 cm long) after solvent evaporation. Using a disposable syringe, the vapor stimuli (3 cm^3^) were blown for 1 s into a constant stream of charcoal-filtered humidified air (500 mL/min) flowing in a stainless steel delivery tube (1 cm i. d.) with the outlet positioned ~1 cm from the antenna. Control (10 μL of hexane) and standard (10 μL of a 100 μg/μL hexanal solution) stimuli were also applied at the beginning of the experiment and after each group of 3 test stimuli. The intervals between stimuli were 1 min. Each concentration of the EO and individual compounds was tested on three antennae from different males and females.

EAG responses were evaluated by measuring the maximum amplitude of negative polarity deflection (-mV) elicited by a stimulus [33]. The amplitude (mV) of the EAG responses to each test stimulus was adjusted to compensate for solvent and/or mechanosensory artefacts by subtracting the mean EAG response of the two nearest hexane controls [34]. The resulting EAG amplitude was corrected according to the reduction of the EAG response to the standard stimulus to compensate for the decrease in the antennal responsiveness during the experiment [35]. Dose–response curves were calculated based on the corrected EAG values. To verify antennal activation, the corrected mean male and female EAG responses to the first dose of the EO or individual compounds were compared to “0” value using the Wilcoxon rank-sum test and regarded as “activated” if significant at *p* = 0.05. Male or female EAG responses to each stimulus tested were compared using Student’s *t*-test for independent samples at *p* = 0.05. Since no significant differences were found between the male and female EAG responses to each test stimulus, individual male and female EAG responses were pooled and analyzed together. For each dose tested, the mean EAG responses of adult insects to the different test stimuli were log-transformed to meet assumptions of normality and submitted to ANOVA followed by Tukey HSD test (*p* = 0.05). Levene’s test was used to assess the homogeneity of variances.

### 2.9. AChE Assay

The anticholinesterase activity of hop EO and its main components was investigated as in Reference [24] by detecting AChE activity photometrically (λ = 412 nm, 25 °C) by means of a Jasco V-570 spectrophotometer (Tokyo, Japan). Briefly, about 0.01 EU of enzyme (EC 3.1.1.7, from *Electrophorus electricus*, SIGMA) were incubated in phosphate buffer (0.1 M, pH 8.00) plus 5,5′dithio bis(2-nitrobenzoic) acid (DTNB, 0.2 mM), either in the absence or in the presence of different aliquots of EO or pure compounds. The reaction was started by the addition of a saturating concentration (2.5 mM) of acetylthiocholine iodide and the rate of absorbance change was obtained as a tangent to the initial part of the progress curve. Results were expressed as % of the control (reaction rate measured in the absence of plant EO/pure compounds). Data were submitted to ANOVA followed by Tukey’s HSD test for mean comparisons.

## 3. Results

### 3.1. EO Characterization

In order to characterize the wild hop ecotype used in this study, the EO composition was investigated by means of GC-MS analysis. In the EO, 29 constituents were identified, accounting for about 98% of the whole EO (Table 1). The main components (92.93%) were: sesquiterpenes (α-humulene, 37.01%; β-caryophyllene, 13.74%; α-selinene 8.70%; β-selinene 6.63%) and monoterpenes (β-myrcene, 26.85%).

### 3.2. Contact Toxicity

The contact toxicities of the EO and its main components against *S. granarius* adults 24 and 48 h after treatment are reported in Table 2. For all samples, mortality increased with the dose increase. Contact mortalities induced by EO were significantly higher than the control, starting from 13.67 and 6.83 µg/adult after 24 and 48 h, respectively (Table 2). EO contact toxicity showed LD_50_ and LD_90_ values of 13.30 and 40.23 µg/adult 24 h after application, slightly decreasing to 11.77 and 36.80 µg/adult after 48 h, respectively (Table 3). Among the individual compounds, β-myrcene, α-humulene, and β-caryophyllene induced significant mortality compared with control starting from 50.06, 27.78, and 56.37 µg/adult, respectively, 24 h after application (Table 2). No significant differences in mortality values between 24 and 48 h after application were found for β-myrcene and β-caryophyllene, whereas for α-humulene the lowest active dose decreased at 13.89 µg/adult at 48 h (Table 2). The highest toxicity was found for α-humulene, which showed LD_50_ and LD_90_ values of 41.87 and 73.51 µg/adult at 24 h and 26.83 and 49.49 µg/adult at 48 h, respectively. LD_50_ and LD_90_ values for β-myrcene were respectively 75.91 and 126.05 µg/adult at 24 h and 73.77 and 123.42 µg/adult 48 h after application (Table 3). The lowest toxicity was calculated for β-caryophyllene since LD_50_ and LD_90_ values were 138.51 and 241.27 µg/adult at 24 h, respectively, with negligible differences between 24 and 48 h after treatment (Table 3).

### 3.3. Inhalation Toxicity

The inhalation toxicities of the EO and the three individual compounds to granary weevil adults are presented in Table 4. Mortality resulting from fumigation with hop EO increased with the dose. The lowest dose showing significant differences compared to the control was 93.33 mg/L air in the absence of food substrate and 122.50 mg/L air in the presence of grains (Table 4). The LC_50_ and LC_90_ values were respectively 132.41 and 198.98 mg/L air in the absence of wheat grains, and 136.37 and 201.48 mg/L air in the presence of food substrate (Table 5).

Regarding individual compounds, in the absence of grains, significant differences in mortality values compared to the control started from 53.40 mg/L air for β-myrcene, whereas such a dose was 124.46 and 120.27 mg/L air for α-humulene and β-caryophyllene, respectively. The presence of wheat grains led to an increase in the lowest effective dose for β-myrcene, 106.80 mg/L air, and α-humulene, 154.09 mg/L air (Table 4). The highest fumigant toxicity was observed for β-myrcene, which showed LC_50_ and LC_90_ values of 72.78 and 116.92 mg/L air in the absence of grains, and 115.78 and 171.42 mg/L air in the presence of food substrate. Without grains, LC_50_ and LC_90_ values were respectively 127.23 and 188.49 mg/L air for α-humulene and 128.15 and 205.67 mg/L air for β-caryophyllene; for both compounds, these values increased (about 20–30%) in the presence of grains (Table 5).

### 3.4. Two-Choice Behavioural Bioassays

**Figure 1:**
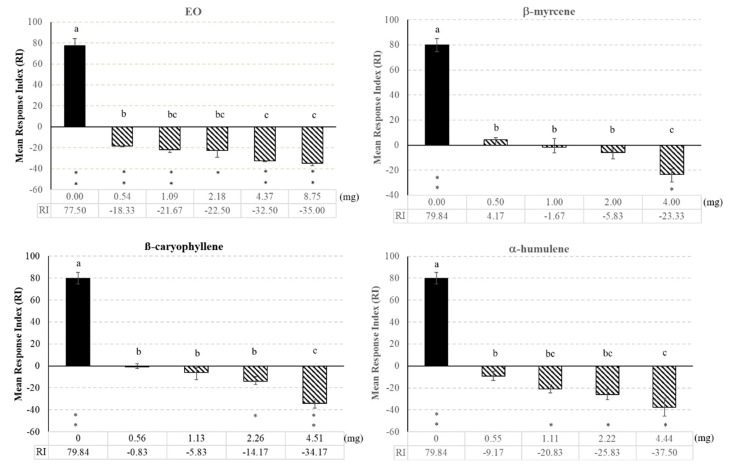
Response Index (RI) of *S. granarius* adults to odours of wheat grains (200 g) alone (black bars) or in the presence of ascending doses of hop EO and its main compounds in two-choice bioassays. For each set of experiments, values with the same letter are not significantly different (*p* < 0.05, Duncan’s MRT test); asterisks indicate significant differences between the number of insects in the treatment and the control (* *p* < 0.05, ** *p* < 0.01; Student’s *t*-test).

Besides toxicity, the hop EO and individual compounds were evaluated in two-choice behavioural bioassays to check for their possible attractive or repellent effects to *S. granarius* adults (Figure 1). Wheat grain odours elicited a highly positive and significant RI, indicating insect attraction. In the dose range tested, the RI to wheat grains was significantly decreased, in a dose dependent manner, by the presence of EO (*F* = 136.08, df = 5, *p* < 0.001), β-myrcene (*F* =76.58, *df* = 4, *p* < 0.001), β-caryophyllene (*F* = 99.90 *df* = 4, *p* < 0.001), and α-humulene (*F* = 73.75, *df* = 4, *p* < 0.001) starting from the first one. Negative and significant RIs, indicating actual repellency, were elicited by EO, α-humulene, β-caryophyllene, β-myrcene at the 0.54, 1.11, 2.26, and 4.00 mg dose, respectively (Figure 1).

### 3.5. EAG

**Figure 2:**
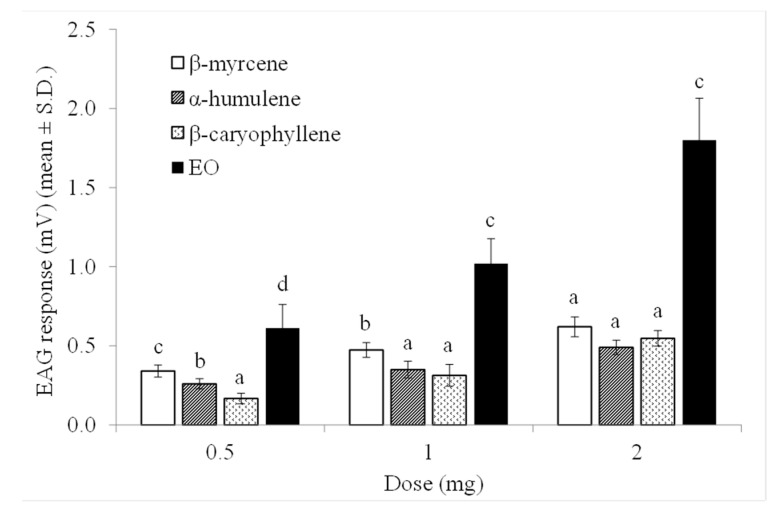
EAG responses of adult *S. granarius* antennae (*n* = 6) to ascending doses of hop EO and its main components. For each dose, different letters indicate significant differences at (*p* < 0.05; Tukey’s HSD test).

The sensitivity of granary weevil antennae toward increasing doses of the hop EO and individual compounds is reported in Figure 2. In the dose range tested, EO and individual compounds elicited measurable (*p* < 0.05 in all Wilcoxon rank-sum test) and dose-dependent EAG responses in both males and females and without significant differences between sexes. The mean EAG response to EO was significantly higher than those to individual compounds at the 0.5 mg (*F* = 68.67, *df* = 3, *p* < 0.001), 1 mg (*F* = 66.90, *df* = 3, *p* < 0.001), and 2 mg (*F* = 194.91, *df* = 3, *p* < 0.001) doses.

### 3.6. Anticholinesterase Activity

**Figure 3:**
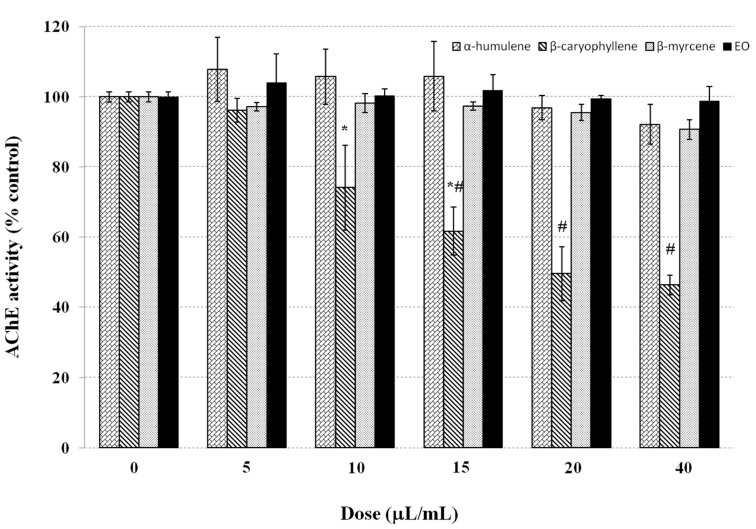
The anticholinesterase activity of hop EO and its main compounds. Mean values (± SE) of AChE activity obtained either in the absence or in the presence of different volumes of α-humulene, β-caryophyllene, β-myrcene, and hop EO. Values (3 for each concentration) were calculated as % of the control (enzyme activity measured in the absence of compound/EO). Density values were 0.889, 0.902, 0.801, and 0.875 g/mL for α-humulene, β-caryophyllene, β-myrcene, and hop EO, respectively. Different symbols indicate a significant difference (P < 0.05, Tukey’s HSD test).

In order to gain a first insight into the toxic mechanism of hop EO and its main compounds, the effects of different doses of EO, *α*-humulene, β-caryophyllene, and β-myrcene on AChE were checked (Figure 3). Negligible anticholinesterase activity was found for all substances in the checked range, with the only exception of β-caryophyllene, which showed a dose-dependent inhibitory effect starting from 9.02 mg/ml (10 µL) and reaching about 50% inhibition at the highest dose checked (36.08 mg/mL, 40 µL) (Figure 3).

## 4. Discussion

The EO extracted from hop cones (female flowers) collected from plants growing wild in the Molise Region (southern Italy) mainly contained α-humulene (37.01%), β-myrcene, (26.85%), and β-caryophyllene (13.74%). A predominant presence of these three compounds was recorded in the fresh EOs of a large number of wild and commercially available hop varieties but in very different proportions [37]. This high variability in the relative abundance of major hop EO components was attributed to intrinsic and extrinsic factors during growth, processing conditions, and the extraction method [38,39].

Despite the extensive use of hop cones as a bittering agent in the brewing industry and clear evidence on the content of compounds with anticancer, antimicrobial, and other positive health effects, the bioactivity of hop cone EO and their main constituents towards stored-product insect pests was little investigated.

Spent hops EO exerted a strong repellent activity against *Rhyzopertha dominica* (F.) and *S. granarius* [40], whilst the EO obtained from cones of a commercial hop variety (cv Cascade) was very effective in killing the invasive mosquito *Aedes albopictus* (Skuse) (Diptera, Culicidae) and freshwater snail *Physella acuta* (Draparnaud) (Mollusca, Physidae) [14].

In this study, good acute contact toxicity was demonstrated for the EO obtained from wild hop cones against the granary weevil adults. In fact, topical application of the EO to adult insects induced high and dose-dependent contact mortality 24 h after treatment. On the basis of the 24 h contact LD_50_ value (13.30 µg/adult), hop EO was less toxic for adult granary weevils than *Carum copticum* L. (0.009 µg/mg body wt) and *Cuminum cyminum* L. (0.016 µg/mg body wt) EOs [41] but more toxic than the EOs of *Hyptis suaveolens* (L.) (LD_50_ = 0.252 µl/adult) and *Hyptis spicigera* Lamarck (LD_90_ = 0.292 µl/adult) [42] and the flower spike EO of *Lavandula angustifolia* Miller (LD_50_ = 58.3 μg/adult) [25], which were all considered as potential sources of bioactive compounds alternative to synthetic insecticides.

This high contact toxicity of hop EOs may be at least in part attributed to its main component α-humulene. Indeed, this compound showed the lowest 24 h contact LD_50_ value (41.87 µg/adult) compared to the other major EO components, β-myrcene (75.91 µg/adult) and β-caryophyllene (138.51 µg/adult). Moreover, 48 h after treatment, the LD_50_ value of α-humulene markedly decreased (26.83 µg/adult), unlike those of β-myrcene and β-caryophyllene that showed only negligible reductions over time. These results are in line with previous researches showing α-humulene as one of the most active compounds contributing to the high contact toxicity of cinnamon and clove EOs to adult granary weevils [43]. It is worth noting that 48 h after treatment, the contact toxicity of *α*-humulene, also known as α-caryophyllene, was almost 5-fold higher than that of its β-isomer, β-caryophyllene, further confirming the importance of isomerism on the biological activity of compounds. Molecules with the same chemical composition but different spatial orientation of their substitutes may have totally different interactions with a specific bind site and, therefore, different biological effects [44].

The 24 h inhalation toxicity of hop EO to adult granary weevils in the absence of wheat grains (LC_50_ = 132.41 mg/L air) was lower than those reported for EOs of many other plants against the same insect pests, including, for instance, *Mentha spicata* L. (about 1 µL/L air) [45], some *Artemisia* species (about 6 µL/L air) [46], *Foeniculum vulgare* Gaertner (27.30 µL/L air) and *Satureja hortensis* L. (52.96 µL/L air) [47], *Salvia leriifolia* (Benth) (79.17 μL/L air) [48], *L. angustifolia* (1.6 mg/L air) [25].

Studies on the toxicity of some essential oils related to their major components showed that the monoterpenes 1,8 cineole [46,49], eugenol [50], camphor [51], and terpinen-4-ol [46] were the most toxic against *S. granarius* and, in some cases, even more toxic than the corresponding EO. None of these compounds were identified in detectable amounts in the hop EO tested in this study, which may account for its poor fumigant toxicity compared to EOs of other aromatic plants. This was also confirmed by the low toxicity of its three major components. Even in the absence of wheat grains, β-myrcene, the most abundant monoterpene in the hop EO, showed the lowest 24 h LC_50_ value (72.78 mg/L air) whereas for the two sesquiterpenes, α-humulene and β-caryophyllene, the LC_50_ values were higher than 100 mg/L air. Moreover, in the presence of wheat grains, an increase in the LC_50_ values was observed for the hop EO and individual compounds tested, suggesting the sorption of compounds to the wheat grains or reduced diffusion of the compound through the interstitial spaces of grains [28,52].

Different from what was recently reported for water and ethanol extracts obtained by hop cones [53], hop EO showed negligible anticholinesterase activity; this strongly suggesting that the inhibition of AChE cannot be the mechanism of action by which EO exerts its toxic activities. This lack of anticholinesterase activity can be explained by EO composition, in which the active compounds reported in Reference [53], as well as others contained in different EOs, proved to have such an effect; α-pinene and 1,8-cineole [54] were completely absent or present at very low levels, as were δ- and γ-cadinene [55]. Indeed, here, β-caryophyllene, but not α-humulene and β-myrcene, showed mild anticholinesterase activity; however, its content (less than 15%), as well as a potential antagonist effect exerted by other compounds [56] resulted in no AChE inhibition by EO. Notice that this latter aspect can be considered as a further advantage for the use of this EO in pest control, the use of insecticides acting on this target being increasingly limited as required by the ongoing search for eco-friendly compounds [57].

The behavioural responses of adult granary weevils to hop EO and their main components were evaluated in two-choice pitfall bioassays in the presence of wheat grains in order to point out possible attractant or repellent effects [27,29]. All stimuli significantly reduced the attractiveness of wheat grain odors to adult insects, starting from the lowest dose tested and in a dose-dependent manner, indicating their effectiveness in disrupting granary weevil orientation to the host substrate.

Among different stimuli, the EO and α-humulene were the most effective repellents since, compared with the other compounds, they elicited a negative and significant RI at lower doses. In electrophysiological recordings, EO and individual compounds evoked EAG dose-dependent responses in adult *S. granarius* antennae, demonstrating the capability of the insect olfactory system to perceive them. An electroantennogram is the summation of receptor potentials evoked by an olfactory stimulus from various olfactory neurons on the insect antennae [58]. Therefore, the higher EAG responses elicited by the EO compared with individual compounds demonstrates that a higher number of receptor neurons were stimulated. This is consistent with the highest repellent activity of the EO and suggests the presence of olfactory neurons specifically tuned to different repellent compounds in the insect antennae.

Considering the toxicity of hop EO and its major compounds to granary weevils, showed by contact and fumigation tests in this study, we can speculate that the decreasing granary weevil attraction to wheat grains might be due to the need to move away from a source of toxic compounds [28].

Such repellent compounds could be used to treat empty stores to flush out hidden infestations before grain storage, to create protective chemical barriers around cereal bulks to disrupt host location by granary weevil adults, or incorporated into packaging materials to prevent insects from entering packaged cereal products [59,60].

The protection of stored cereals and derived products with EOs or their active components is one of the most promising research areas for the development of new and sustainable options to be implemented in integrated pest management strategies. However, proper formulations of these substances must be developed to overcome some drawbacks such as the need to guarantee a long-lasting release of the active ingredients at a constant and effective concentration and to limit the impact of their strong odours on food products.

## 5. Conclusions

This study clearly shows that wild hop EO possesses an interesting biological activity against the pest *S. granarius*, which can be in part attributed to α-humulene, β-caryophyllene, and β-myrcene, the three most abundant constituents of the EO. Thus, the high contact and the moderate inhalation toxicities, together with the negligible anticholinesterase of EO and its compounds, makes wild hop an excellent low-cost resource for the production of eco-friendly alternative to synthetic insecticides. In addition, hop EO showed a good repellent activity able to disrupt *S. granarius* orientation to a very attractive host substrate, indicating also a possible application as an insect behaviour-modifying compound to prevent granary weevil infestation of stored cereals and derived food products. Therefore, the use of hop cones for purposes different from beer production is increasingly gaining interest.

## Figures and Tables

**Table 1:** Chemical composition (%) of hop EO.

COMPOUNDS	R.T.	RI_Exp_^(1)^	RI_Lit_^(2)^	% TOTAL ± S.E. ^(3)^
β-Pinene	12.54	932	932	0.40 ± 0.04
β-Myrcene	13.20	990	988	26.85 ± 4.89
Methyl heptanoate	14.64	1023	1021	0.21 ± 0.05
Methyl 6-methyl-heptanoate	16.50	1072	1070	0.14 ± 0.03
2-Nonanone	17.40	1088	1087	0.31 ± 0.05
β-Linalool	18.17	1097	1095	0.72 ± 0.08
Methyl octanoate	19.33	1124	1123	0.20 ± 0.04
2-Decanone	22.47	1188	1190	0.23 ± 0.04
3-Octen-1-ol, acetate	26.79	1288	1290	0.24 ± 0.03
Methyl dec-4-enoate	26.99	1290	1289	1.05 ± 0.15
2-Undecanone	27.21	1293	1293	13.63 ± 2.84
Methyl decanoate	28.28	1325	1326	0.13 ± 0.02
α-Copaene	30.57	1375	1374	0.21 ± 0.02
β-Panasinsene	30.98	1380	1381	0.57 ± 0.07
2-Dodecanone	31.26	1390	1388	0.16 ± 0.02
β-Caryophyllene	32.47	1418	1417	13.74 ± 1.89
β-Copaene	32.78	1430	1430	0.26 ± 0.03
γ-Elemene	32.93	1435	1434	0.59 ± 0.06
α-Humulene	33.84	1460	1462	37.01 ± 5.31
4,5-di-epi-Aristolochene	34.10	1471	1471	0.37 ± 0.06
γ-Gurjunene	34.50	1472	1475	0.60 ± 0.06
γ-Selinene	34.66	1474	1476	1.80 ± 0.24
β-Selinene	35.14	1490	1489	6.63 ± 0.12
2-Tridecanone	35.31	1495	1495	1.09 ± 0.18
α-Selinene	35.50	1499	1498	8.70 ± 0.28
(*E*,*E*)-α-Farnesene	35.81	1507	1505	0.69 ± 0.07
γ-Cadinene	36.14	1515	1513	0.75 ± 0.06
δ-Cadinene	36.49	1524	1522	1.38 ± 0.21
Selina-3,7(11)-diene	37.20	1543	1545	0.72 ± 0.06

^(1)^ RI_Exp_ = Determined linear retention index against mixture of *n*-alkanes (C8-C20) on HP-5MS column. ^(2)^ RI_Lit_ = Linear retention index from the literature [36]. ^(3)^ *N* = 3 replicates.

**Table 2:** Mortality (%) of *S. granarius* adults registered 24 and 48 h after topical application of different concentrations of hop EO, β-myrcene, α-humulene, and β-caryophyllene. For each exposure time, values in the same column followed by the same letter are not significantly different (*p* ≤ 0.05, Tukey’s HSD test).

Expos. Time (h)	EO		β-Myrcene		α-Humulene		β-Caryophyllene	
	Dose (µg/Adult)	% Mortality (Mean ± S.E.)	Dose (µg/Adult)	% Mortality (Mean ± S.E.)	Dose (µg/Adult)	% Mortality (Mean ± S.E.)	Dose (µg/Adult)	% Mortality (Mean ± S.E.)
24	109.37	100.00 ± 0.00 a	200.25	100.00 ± 0.00 a	222.25	100.00 ± 0.00 a	225.50	87.50 ± 3.66 a
	54.68	93.33 ± 4.22 ab	100.12	70.00 ± 3.78 b	111.12	97.50 ± 2.50 a	112.75	35.00 ± 3.27 b
	27.34	76.67 ± 6.15 bc	50.06	37.50 ± 4.53 c	55.56	77.50 ± 2.50 b	56.37	17.50 ± 2.50 c
	13.67	60.00 ± 5.16 c	25.03	7.50 ± 3.66 d	27.78	32.50 ± 5.26 c	28.19	10.00 ± 3.78 cd
	6.83	16.67 ± 6.15 d	12.51	5.00 ± 3.27 d	13.89	12.50 ± 5.26 d	14.09	10.00 ± 3.78 cd
	3.42	6.67 ± 4.22 d	0.00	0.00 ± 0.00 d	0.00	0.00 ± 0.00 d	0.00	0.00 ± 0.00 d
	0.00	0.00 ± 0.00 d						
	*F*	90.40	*F*	169.44	*F*	170.80	*F*	104.28
	*d.f.*	6	*d.f.*	5	*d.f.*	5	*d.f.*	5
	*p*	< 0.001	*p*	< 0.001	*p*	< 0.001	*p*	< 0.001
48	109.37	100.00 ± 0.00 a	200.25	100.00 ± 0.00 a	222.25	100.00 ± 0.00 a	225.50	87.50 ± 3.66 a
	54.68	96.66 ± 3.33 ab	100.12	72.50 ± 3.66 b	111.12	100.00 ± 0.00 a	112.75	40.00 ± 6.55 b
	27.34	76.67 ± 6.15 bc	50.06	37.50 ± 4.53 c	55.56	97.50 ± 2.50 a	56.37	20.00 ± 3.78 c
	13.67	60.00 ± 5.16 c	25.03	10.00 ± 3.78 d	27.78	42.50 ± 2.50 b	28.19	10.00 ± 3.78 cd
	6.83	30.00 ± 6.83 d	12.51	5.00 ± 3.27 d	13.89	27.50 ± 7.50 b	14.09	10.00 ± 3.78 cd
	3.42	6.67 ± 4.22 e	0.00	0.00 ± 0.00 d	0.00	3.33 ± 3.33 c	0.00	0.00 ± 0.00 d
	0.00	3.33 ± 3.33 e						
	*F*	76.18	*F*	170.04	*F*	108.11	*F*	62.72
	*d.f.*	6	*d.f.*	5	*d.f.*	5	*d.f.*	5
	*p*	< 0.001	*p*	< 0.001	*p*	< 0.001	*p*	< 0.001

**Table 3:** Contact toxicity of hop EO, β-myrcene, α-humulene, and β-caryophyllene against *S. granarius* adults 24 and 48 h after topical application.

Expos. Time (h)		EO	β-Myrcene	α-Humulene	β-Caryophyllene
24	LD_50_ (95% CL, µg/adult)	13.30 (10.66 – 16.44)	75.91 (66.42 – 88.26)	41.87 (27.89 – 66.07)	138.51 (120.41 – 161.77)
	LD_90_ (95% CL, µg/adult)	40.23 (30.53 – 59.99)	126.05 (109.63 – 151.83)	73.51 (54.58 – 138.17)	241.27 (209.65 – 288.77)
	regression equation	*y* = 2.666*x* – 2.996	*y* = 0.026*x* – 1.94	*y* = 0.041*x* – 1.696	*y* = 0.013*x* – 1.73
	χ^2^	2.04	5.05	11.09	3.61
48	LD_50_ (95% CL, µg/adult)	11.77 (9.35 – 16.60)	73.77 (64.48 – 85.79)	26.83 (22.57 – 31.77)	134.72 (116.68 – 157.92)
	LD_90_ (95% CL, µg/adult)	36.80 (27.74 − 55.73)	123.42 (107.33 – 148.57)	49.49 (42.62 – 60.49)	238.69 (207.68 – 288.05)
	regression equation	*y* = 2.588*x* – 2.771	*y* = 0.026*x* – 1.90	*y* = 0.057*x* – 1.518	*y* = 0.012*x* – 1.64
	χ^2^	1.42	4.04	2.57	3.62

**Table 4:** Mortality (%) of *S. granarius* adults, in the absence or presence of wheat grains (200 g), 24 h after fumigation with different concentrations of hop EO, β-myrcene, α-humulene, and β-caryophyllene. For each exposure time, values in the same column followed by the same letter are not significantly different (*p* ≤ 0.05, Tukey’s HSD test).

Wheat Grains	EO		β-Myrcene		A-Humulene		β-Caryophyllene	
	Dose (mg/L)	% Mortality (Mean ± S.E.)	Dose (mg/L)	% Mortality (Mean ± S.E.)	Dose (mg/L)	% Mortality (Mean ± S.E.)	Dose (mg/L)	% Mortality (Mean ± S.E.)
**NO**	210.00	100.00 ± 0.00 a	146.85	100.00 ± 0.00 a	183.73	100.00 ± 0.00 a	210.47	100.00 ± 0.00 a
	180.83	76.67 ± 6.67 b	106.80	73.33 ± 3.33 b	154.09	63.33 ± 6.67 b	165.37	66.67 ± 8.82 b
	151.67	63.33 ± 3.33 b	53.40	46.67 ± 3.33 c	124.46	43.33 ± 3.33 c	120.27	26.67 ± 6.67 c
	122.50	36.67 ± 3.33 c	26.70	6.67 ± 3.33 d	94.83	13.33 ± 3.33 d	60.13	23.33 ± 3.33 cd
	93.33	20.00 ± 5.77 cd	13.35	3.33 ± 3.33 d	47.41	10.00 ± 5.77 d	30.07	13.33 ± 8.82 cd
	46.67	10.00 ± 5.77 de	6.68	3.33 ± 3.33 d	23.71	0.00 ± 0.00 d	15.03	3.33 ± 3.33 cd
	23.33	6.67 ± 6.67 de	3.34	0.00 ± 0.00 d	11.85	6.67 ± 3.33 d	7.52	0.0 ± 0.0 d
	11.67 *	0.00 ± 0.00 d	0.00	0.00 ± 0.00 d	5.93 *	0.0 ± 0.0 d	3.76	0.0 ± 0.0 d
	0.00	0.00 ± 0.00 e			0.00	0.0 ± 0.0 d	0.00	0.0 ± 0.0 d
	*F*	*86.69*	*F*	*113.34*	*F*	*113.34*	*F*	*49.81*
	*d.f.*	*12*	*d.f.*	*7*	*d.f.*	*11*	*d.f.*	*8*
	*P*	*<0.001*	*P*	*<0.001*	*P*	*<0.001*	*P*	*<0.001*
**YES**	210.00	100.00 ± 0.00 a	186.90	100.00 ± 0.00 a	213.36	100.00 ± 0.00 a	240.53	100.00 ± 0.00 a
	180.83	73.33 ± 8.82 b	146.85	63.33 ± 6.67 b	183.73	56.67 ± 8.82 b	210.47	63.33 ± 3.33 b
	151.67	60.00 ± 5.77 b	106.80	40.00 ± 5.77 c	154.09	26.67 ± 3.33 c	165.37	36.57 ± 3.33 c
	122.50	36.67 ± 3.33 c	53.40	13.33 ± 6.67 d	124.46	13.33 ± 3.33 cd	120.27	20.00 ± 5.77 d
	93.33	16.67 ± 3.33 d	26.70	3.33 ± 3.33 d	94.83	10.00 ± 5.77 cd	60.13	6.67 ± 3.33 e
	46.67	10.00 ± 5.77 d	13.35	0.00 ± 0.00 d	47.41	6.67 ± 6.67 d	30.07	0.00 ± 0.00 e
	23.33	3.33 ± 3.33 d	6.68	0.00 ± 0.00 d	23.71	3.33 ± 3.33 d	15.03	0.00 ± 0.00 e
	11.67 *	0.00 ± 0.00 d	3.34	0.00 ± 0.00 d	11.85 *	0.00 ± 0.00 d	7.52 *	0.00 ± 0.00 e
	0.00	0.00 ± 0.00 d	0.00	0.00 ± 0.00 d	0.00	0.00 ± 0.00 d	0.00	0.00 ± 0.00 e
	*F*	84.02	*F*	87.94	*F*	61.05	*F*	177.70
	*d.f.*	12	*d.f.*	8	*d.f.*	12	*d.f.*	9
	*p*	< 0.001	*p*	< 0.001	*p*	< 0.001	*p*	< 0.001

* lower doses showing no mortality were omitted in the table for its clarity.

**Table 5:** Fumigant toxicity of hop EO, β-myrcene, α-humulene, and β-caryophyllene in the absence or presence of wheat grains (200 g) against S. granarius adults 24 h after exposure.

WG		EO	β-Myrcene	α-Humulene	β-Caryophyllene
**NO**	LC_50_ (95% CL, mg/L)	132.41 (121.89 – 143.89)	72.78 (63.91 – 83.35)	127.23 (108.94 – 150.14)	128.15 (103.8 ± 5 – 161.07)
	LC_90_ (95% CL, mg/L)	198.98 (183.32 – 219.86)	116.92 (103.50 – 135.95)	188.49 (162.78 – 234.61)	205.67 (170.39 – 272.64)
	regression equation	*y* = 0.019*x* – 2.55	*y* = 0.029*x* – 2.11	*y* = 0.021*x* – 2.66	*y* = 0.017*x* – 2.12
	χ^2^	10.34	9.17	23.55	16.07
**YES**	LC_50_ (95% CL, mg/L)	136.37 (125.88 – 147.79)	115.78 (104.79 – 127.91)	164.95 (145.95 – 190.06)	172.60 (159.15 – 187.08)
	LC_90_ (95% CL, mg/L)	201.48 (185.91 – 222.51)	171.42 (155.90 – 193.13)	234.39 (205.43 – 289.39)	244.78 (225.37 – 272.90)
	regression equation	*y* = 0.020*x* – 2.68	*y* = 0.023*x* – 2.67	*y* = 0.018*x* – 3.04	*y* = 0.018*x* – 3.06
	χ^2^	8.64	5.61	23.02	9.88
